# A Low-Interaction Automatic 3D Liver Segmentation Method Using Computed Tomography for Selective Internal Radiation Therapy

**DOI:** 10.1155/2014/198015

**Published:** 2014-07-03

**Authors:** Mohammed Goryawala, Seza Gulec, Ruchir Bhatt, Anthony J. McGoron, Malek Adjouadi

**Affiliations:** ^1^Department of Electrical Engineering at the Florida International University, Miami, FL 33174, USA; ^2^Herbert Wertheim College of Medicine at the Florida International University, Miami, FL 33174, USA; ^3^Biomedical Engineering Department at the Florida International University, Miami, FL 33174, USA

## Abstract

This study introduces a novel liver segmentation approach for estimating anatomic liver volumes towards selective internal radiation treatment (SIRT). The algorithm requires minimal human interaction since the initialization process to segment the entire liver in 3D relied on a single computed tomography (CT) slice. The algorithm integrates a localized contouring algorithm with a modified k-means method. The modified k-means segments each slice into five distinct regions belonging to different structures. The liver region is further segmented using localized contouring. The novelty of the algorithm is in the design of the initialization masks for region contouring to minimize human intervention. Intensity based region growing together with novel volume of interest (VOI) based corrections is used to accomplish the single slice initialization. The performance of the algorithm is evaluated using 34 liver CT scans. Statistical experiments were performed to determine consistency of segmentation and to assess user dependency on the initialization process. Volume estimations are compared to the manual gold standard. Results show an average accuracy of 97.22% for volumetric calculation with an average Dice coefficient of 0.92. Statistical tests show that the algorithm is highly consistent (*P* = 0.55) and independent of user initialization (*P* = 0.20 and Fleiss' Kappa = 0.77 ± 0.06).

## 1. Introduction

According to the American Cancer Society, 1 in every 94 men and 1 in every 212 women born are susceptible to liver cancer through their life time [1, 2]. Liver cancer treatment which delivers maximum radiation dose to the tumor and minimum toxicity to the surrounding healthy tissue has been one of the major challenges in clinical practice [3, 4]. Selective internal radiation therapy (SIRT) with Yttrium-90 (Y-90) microspheres is an effective technique for liver-directed therapy [5]. SIRT dosimetry, however, requires accurate determination of the relative functional tumor(s) volume(s) with respect to the anatomical volumes of the liver in order to estimate the Y-90 microsphere dose to be delivered to the tumor [6, 7]. Clinically, accurate liver volume determination is most often accomplished through tedious manual segmentation of the entire computerized tomography (CT) scan, a task greatly dependent on the skill of the operator. Automatic/semiautomatic approaches are thus geared towards segmenting and determining the volume of the liver accurately while facilitating the operational process from a clinician/physician's viewpoint.

Segmentation is an important preprocessing step in many image processing applications including complex tasks such as brain segmentation from MR images [8, 9], lung segmentation from CT images [10–12], and other types of medical image analysis [13]. Current automatic and semiautomatic procedures for liver segmentation are based on techniques that rely on (1) shape constrained segmentation using heuristic approaches [14–16], including local shape models [17–19], atlas based techniques [20–24], or nonlinear models [25, 26]; (2) rule-based segmentation [27–29]; (3) gradient vector flow [30–32]; and (4) two- or three-dimensional region growing methods [33–36]. Although these techniques offer highly accurate results, the algorithms need to accommodate for varying protocols, data from different sources, artifacts, and the presence of pathological structures such as tumors [37, 38]. Also, algorithms used in cases of liver segmentation tend to be difficult to operate for a person with limited amount of know-how given the subtle imaging conditions one has to contend with and the skills needed in delineating liver from surrounding organs. In an earlier study, we presented a similar approach to liver segmentation that required initialization in multiple slices across a dataset [39].

The main contributions of this study include the following: (1) establishing a hybrid approach which utilizes the k-means based segmentation algorithm coupled with a new application of a localized contouring algorithm specifically for CT datasets, based on local regional thresholds defined around the point of interest in terms of relative radio density; (2) developing a novel process for liver volume calculations based on a single slice initialization using a multistep approach that is shown to yield high accuracy; and (3) ensuring that the developed technique is fairly independent of the user performing the initialization process.

## 2. Methods

**Figure 1:**
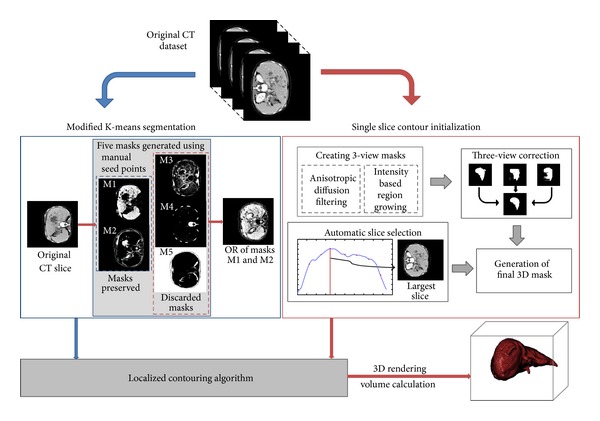
Modular block diagram of the main steps for liver segmentation and volume calculation.

Figure 1 illustrates the block diagram with the steps that make up the proposed algorithm. The following sections describe in detail the various modules of the algorithm, namely, the modified k-means algorithm (Section 2.1), the single slice contour initialization (Section 2.2), the localized region growing (Section 2.3), and the three-dimensional image rendering process (Section 2.4).

Sections 2.5 and 2.6 outline the evaluation of the results of the algorithm and the statistical analysis carried out to demonstrate the independence of the algorithm to user initialization, respectively.

### 2.1. Modified k-Means Algorithm for Liver Segmentation

The first step for segmenting liver tissue from CT involves the extraction of organs and structures, which can be easily segmented out. To accomplish this step, a modified version of the well-known k-means algorithm adopted for abdominal CT is utilized [45]. The user is presented with the central image of the dataset housing the entire liver, as it possesses all the needed features for segmentation. The five regions identified on each CT slice are the following: (1) liver, (2) surrounding organs, (3) peripheral muscles, (4) ribs/spinal cord, and (5) outside of the body.

**Figure 2:**
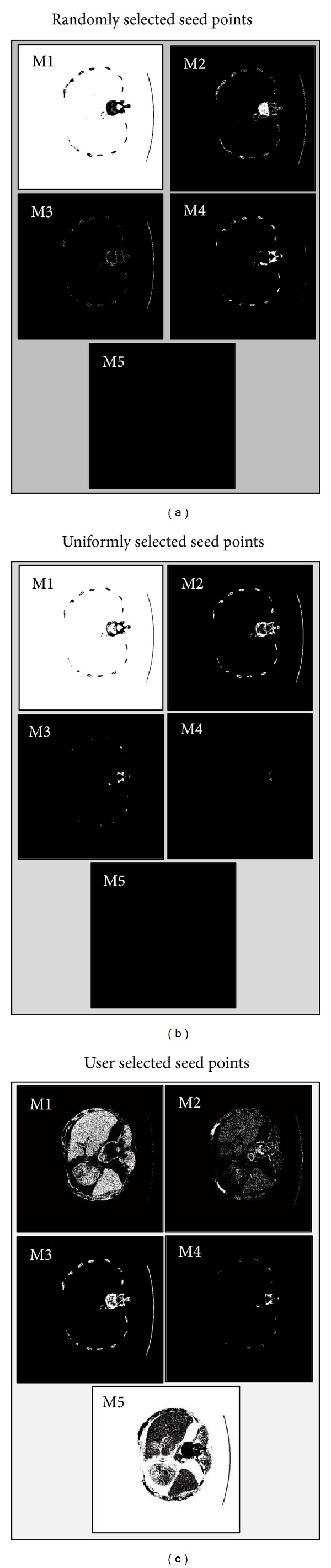
Importance of selection of seeds points for the k-means algorithm; (a) 5 masks M1–M5 obtained using random selected seed points, (b) 5 masks M1–M5 obtained using uniformly selected seed points, and (c) 5 masks M1–M5 obtained using user selected seed points. Results show that a user identification of the 5 regions as outlined in this study results in a better demarcation of liver tissue.

Selection of the seed points for the k-means algorithm is an important first task. A random selection of seeds for the clusters as shown in Figure 2 results in the generation of incorrect masks. This is due to the clusters being formed around those random seeds, which range from −1024 HU to 3000 HU for a typical CT slice. Since the liver is typically seen in the −40 HU to 180 HU range, random selection of seeds is the least likely to achieve the needed segmentation.

In the case of uniformly selected points in the range of the CT acting as seeds, the centroids are found to be around −1024 HU, −205 HU, 614 HU, 1433 HU, and 2252 HU. These seeds also fail to segment the regions containing the liver from the image as seen in Figure 2. An alternative would be to use uniformly selected seed points in the −40 HU to 180 HU liver range. Empirically, however, such a selection of seeds yields centroids too close to one another for suitable segmentation. Also a large number of points below −40 HU and above 180 HU were misclassified.

Hence, the choice for user selected points to act as the seeds for each of the aforementioned five masks is shown in Figure 2 for any given slice. These seed points clearly differentiate the various organs of interest as compared to the other two methods of seed selection where the liver region is not at all visible.

Also segmentation of each image is replicated 3 times to achieve better results, where the intersection of the results of the 3 runs for each image slice is taken to obtain the final segmentation. Lastly, the modified k-means is operated on a so called “online update mode” where the sum of distances is calculated with the movement of every pixel to a different cluster. Although this step is slightly more time consuming than when using a batch update, higher accuracies are, however, guaranteed since the local minima of the distance function can be calculated more precisely.

Empirical results showed that in some cases the liver was not entirely reflected through mask M1 alone due to inhomogeneous intensity distribution across the entire liver region but does show a spillover into the adjoining M2 mask (surrounding organs). Thus, in order to avoid underestimating the liver tissue, masks M1 and M2 are combined (ORed (+)) to obtain the final mask (M_*F*_) as shown in the following:

(1) $$\begin{array}{c} {\text{M}}_{F}=\text{M}1+\text{M}2. \end{array}$$

This final mask (M_*F*_) generated for each slice is applied on the CT images for a rough estimation of the liver region.

The modified k-means segmentation consists of the following steps.

1. Load a 3D liver dataset from DICOM images.
2. Present to the user the central slice of the liver. This central slice is chosen to be representative of the different intensity structures in the abdomen.
3. User selects 5 points in the central slice belonging to (1) liver, (2) surrounding organs, (3) peripheral muscles, (4) ribs/spinal cord, and (5) outside of the body.
4. Carry out a modified k-means segmentation minimizing within-cluster sum of square in the online update mode with 3 repetitions for more reliable segmentation results. For the k-means segmentation, a maximum of 100 iterations with a stopping condition of less than 1e-5 difference in across clusters is used. User selected seed points are used as initial centroids for clustering purposes.
5. Combine clusters from M1 and M2 to obtain a rough estimation of the liver region.

### 2.2. Single Slice Contour Initialization

Following the coarse segmentation obtained by the modified k-means algorithm, a localized contouring algorithm is adopted for refining the segmentation to obtain accurate volumetric estimations of the liver. Localized contouring is based on developing a dynamically growing contour around the liver using the local properties around point under consideration rather than the entire statistics of the image. Localized region growing algorithms are more robust than contouring algorithms which are based on global energies not suitable for segmenting heterogeneous objects like the liver [46].

However, a significant issue of concern in performing any contouring algorithm is the initialization of the contour, especially for the case of liver segmentation, where other organs in close proximity have similar intensity distributions. A prior study by our group attempted to resolve this issue by invoking the user to initialize multiple slices across the 3D data set [39, 47]. However, in an effort to reduce user interaction to a minimum, while maintaining high segmentation accuracy, the initialization process in this new study relies on a single CT slice. The three-step single slice initialization procedure is outlined in Section 2.2.1 through 2.2.3.

#### 2.2.1. Automated Intensity Based Region Growing

Intensity-based region growing works by accepting a seed point and expanding the region of interest based on the intensity of the neighboring region. Thus, both the selection of the seed point and the intensity threshold for inclusion of voxels in the region are two important features of the region growing process. The user selects a single point belonging to the liver mass in the first and last slice. These two points act as the endpoints of a seed-line that contains the seed points for the region growing algorithm. The intersection point of the seed-line and a given image slice is used as the seed point for the region growing algorithm for that particular slice.

Linear interpolation was used to generate the intersection points for the various slices, based on the assumption that the structure of the liver should not change abruptly from slice to slice. Empirical evidence supports this assertion. Nonetheless, recognizing the inherent tendency of inhomogeneity in CT images from scan to scan, a region growing threshold is devised in terms of the standard deviation of the image under consideration. Noting that intensities considered for the algorithm are actual Hounsfield units, a threshold equivalent to 5% of the standard deviation of the image is used.

#### 2.2.2. Volume of Interest (VOI) Based Correction

The region growing algorithm based on intensity absorbs some portions of neighboring regions like the spleen and the stomach. The correction algorithm is based on the idea that a voxel is considered as belonging to liver tissue in the 3D space only if it is marked by the automated intensity based region growing in at least two of the three views (axial, transverse, and coronal).

**Figure 3:**
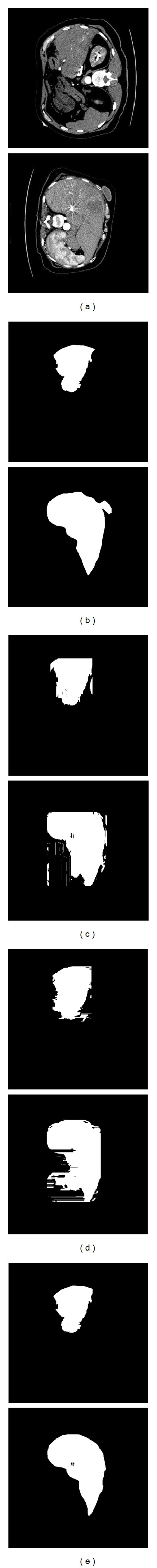
Examples of volume of interest (VOI) based correction: (a) original slice, (b) axial, (c) sagittal, (d) transversal, and (e) VOI corrected masks. It can be observed that VOI corrected liver masks as shown in (e) are able to eliminate false positives.

The 3D corrected mask is generated using the following equation:

(2) $$\begin{array}{c} M_{\text{Correct}}^{3\text{D}}=M_{\text{Cor}}^{3\text{D}}\cdot M_{\text{Sag}}^{3\text{D}}+M_{\text{Sag}}^{3\text{D}}\cdot M_{\text{Tran}}^{3\text{D}}+M_{\text{Tran}}^{3\text{D}}\cdot M_{\text{Cor}\;}^{3\text{D}}, \end{array}$$

where *M*_Correct_^3D^ denotes the 3D corrected mask and *M*_Cor_^3D^, *M*_Sag_^3D^, and *M*_Tran_^3D^ are the 3D axial, sagittal, and transversal masks, respectively. The “·” operator suggests a pixel-by-pixel AND operation and the “+” operator suggests a pixel-by-pixel OR operation. The results of such a correction process are exemplified in Figure 3 where it can be seen that the corrected masks as obtained in part (e) eliminated most false positives.

#### 2.2.3. Automatic Largest Liver Slice Selection

The final step in the initialization process is the masking of the results obtained from the aforementioned region growing algorithm with the mask of the largest slice. In the context of the algorithm, the largest slice is defined as the slice in which the liver is seen in its largest extent. To determine which of the slices is the largest, an automatic largest slice selection (ALSS) algorithm developed in house is utilized. The ALSS algorithm begins by first dividing the entire image into blocks of 16 × 16 pixels.

**Figure 4:**
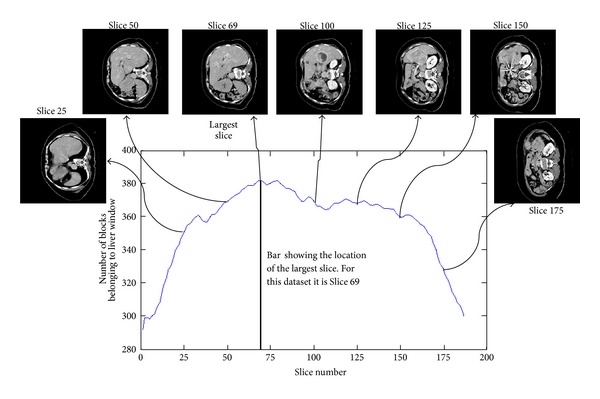
Details of the ALSS algorithm. A typical profile curve is shown with some of the corresponding slices used to determine it. The vertical black bar indicates the location of the largest slice.

Inside each block, the pixels that fall within the abdominal CT window of −40 HU to 180 HU were counted [34]. A block was marked as being part of the liver region if at least more than half of the pixels were found in the abdominal CT window as described in the following:

(3) $$\begin{array}{c} \text{block}\;\text{marked}=\{\begin{array}{cc} 1 & \text{if}\;\frac{n}{N}\geq 0.5\\ 0 & \text{otherwise}, \end{array} \end{array}$$

where *n* and *N* are the number of pixels in the abdominal window and the total number of pixel elements in the block, respectively.  Figure 4 shows a typical profile curve generated for dataset 1, which displays the number of blocks belonging to the liver window as a function of the slice number and the slice number for the estimated largest slice of the dataset.

The slice marked by the ALSS algorithm is presented to the user to set the boundaries of the liver in that slice. The process generates a mask which shows the largest slice of the dataset. The mask is applied to all the slices of the dataset to exclude any portions which do not belong to the liver region. This step is essential since the region growing algorithm sometimes overextracts the liver region by absorbing some regions that belong to the duodenum and the spleen regions.

On completion of this final step of the initialization process, which is essentially a VOI-based correction, the localized region contouring algorithm can be applied to the images extracted through the k-means algorithm. Thus, the completion of the entire initialization process results in individual slice masks generated for every slice of the dataset containing any portion of the liver on which the localized contouring algorithm is implemented.

The steps of the region growing algorithm for contour initialization are given below.

1. Present to user the first and last slices of the liver volume to select a single point in the liver mass in each slice.
2. Construct the seed line joining the user selected points using linear interpolation to span the slices of the liver dataset.
3. Determine points of intersection of the slices and seed line to derive seed points for each slice of the dataset.
4. Perform region growing beginning at user selected seed point by assimilating all points in a 3 × 3 neighborhood that are within the 5% threshold as described in Section 2.2.1.
5. Repeat steps 1–4 for axial, transversal, and sagittal images.
6. Perform VOI-based correction as per (2).
7. Determine the slice with the largest liver mass using the ALSS algorithm as per (3) to mask out any false positives from the region growing step.

### 2.3. Localized Region Growing

Once the initialization for all the slices of the dataset is obtained interactively, a modified localized region-based active contouring algorithm is employed. The details of the contouring algorithm have been presented in an earlier study [47]. Localized region growing algorithms are more robust than contouring algorithms which are based on global energies for segmenting heterogeneous objects like the liver [46]. Localized contouring algorithms that segment the image based on properties of a small region around a point of interest rather than the entire image tend to segment the liver with better accuracy.

The stepwise details of the localized contouring algorithm are given below.

1. The VOI and ALSS corrected mask for each slice is fed to the localized contouring algorithm as an initialization.
2. The contour is modeled as a smoothened approximation of the Heaviside function and fitted to reduce the Chan-Vase energy approximations of the interior and exterior of the contour [46, 47].
3. For localization effect, a region of radius *α* is selected about the point of interest to restrict energy calculations to a localized region.
4. Update contour for a maximum of 100 iterations or until the difference between the Chan-Vase energies of the interior and exterior of the contour do not differ by more than 1e-6.
5. Designate the interior of the contour as the final segmented liver and calculate the volume.

### 2.4. Three-Dimensional Image Rendering

The 3D datasets are rendered using cost-effective third party software called ScanIP developed by Simpleware Ltd. based in the United Kingdom. The software renders the segmented dataset in 3D space and offers the possibility for the physician to view/edit/correct the rendered liver if deemed necessary. The software also calculates the volume of the liver by determining the number of voxels that are marked as being within the liver region by the segmentation algorithm. The only inputs fed to the software are the segmented dataset and the original resolution of the CT datasets. The calculated volumes are in milliliters (mL).

### 2.5. Segmentation Evaluation

Thirty-four datasets acquired using a combined PET/CT system (GE Discovery LS) are used for testing the proposed algorithm. The scanning parameters were 140 kVp, 80 mA, 0.5 s rotation time, and 512 × 512-pixel matrix. The images were provided in the DICOM format. Pixel sizes ranged from 0.54 to 0.97 mm. For each dataset, a stack of 132–377 slices was acquired. The data are of patients who underwent treatment for Hepatic cancer with multiple tumors in the liver. A majority of these patients suffered from adenocarcinoma of the colon showing multiple metastatic tumors in liver with hepatic and extrahepatic involvement.

In order to validate the segmentation results obtained by the algorithm, the volumes of the extracted livers were compared to manually calculated volumes. The manual calculation was done by a clinical expert at Jackson North Medical Center and is considered as the gold standard for the comparison, which is routinely the suggested method [38].

Also, to access the performance of the algorithm the Dice coefficients between the segmentation obtained manually and by the algorithm are computed using:

(4) $$\begin{array}{c} k\left(I_{\text{ref}},I\right)=\frac{2\left|I_{\text{ref}}\cap I\right|}{\left|I_{\text{ref}}\right|+\left|I\right|}, \end{array}$$

where *I*_ref_ is the reference segmentation obtained by manual method and *I* is the segmentation obtained by the proposed algorithm. The Dice coefficient is routinely used in image segmentation as a validation measure of the pixel-by-pixel matching between the segmented image and the gold standard [48].

### 2.6. Statistical Evaluation

To validate the consistency of the algorithm in segmenting the liver, 3 runs are carried out whereby each liver is segmented 3 times and the absolute errors obtained in the calculation of the volumes is reported. To provide a statistical measure of consistency in the results, 1-way analysis of variance (ANOVA) is carried out. The ANOVA is used to determine if the various runs have a significant impact on the absolute errors and hence the segmentation accuracy.

In order to determine if the user initialization of the segmentation has an impact on the results, 3 different users with clinical experience and with medical or engineering backgrounds were asked to initialize the algorithm. In order to provide a profile of the users selected for the study, each user was asked to rate themselves on a scale of 1 to 5 for their knowledge of liver anatomy and algorithm/program development.  Table 1 provides the profiles of the different users selected for task initialization. One-way ANOVA test was carried out on the errors obtained after the different users initialized the segmentation process.

To gauge the interrater variability, the Fleiss' kappa [49] statistics were used to explain the interrater variations for each slice of each of the datasets used in the study. Fleiss' kappa assesses the reliability of agreement between a fixed number of raters/users (in this case 3) when assigning categorical ratings to a number of items or classifying items, in this case as foreground (liver) or background. To accomplish the randomness of the decisions on each pixel of a slice of the liver dataset was calculated from the different segmentations produced by the initialization of different individuals [50].

The Fleiss' Kappa measure *κ* is generally calculated as

(5) $$\begin{array}{c} \kappa =\frac{\overset{-}{P}-\overset{-}{P_{e}}}{1-\overset{-}{P_{e}}}, \end{array}$$

where $\overset{-}{P}$ is the relative observed pixel-wise agreement among the segmentations obtained from various users and *P*_*e*_ is the hypothetical probability of chance agreement. Since $1-\overset{-}{P_{e}}$ demonstrates the degree of agreement that is attainable due to chance, $\overset{-}{P}-\overset{-}{P_{e}}$  gives the degree of agreement actually achieved among the various segmentations.

**Figure 5:**
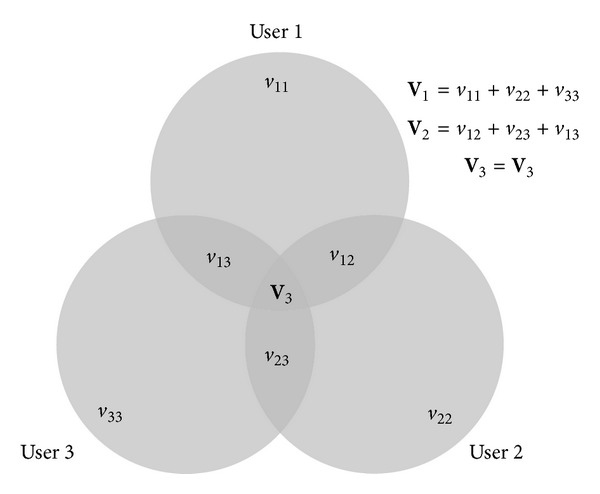
Pictorial representation of calculation of Fleiss' Kappa. **V**_3_: volume delineated by all three observers (intersection); **V**_2_: sum of the three volumes delineated by two observers; **V**_1_: sum of the three volumes delineated by only one observer.

For estimation of the Fleiss' Kappa in imaging applications, the method outlined by Rücker et al. was adopted [51]. Accordingly, the Fleiss' Kappa for the 3 users was estimated as

(6) $$\begin{array}{c} \kappa =\frac{V_{2}-{3V}_{3}}{V_{1}+2V_{2}+3V_{3}}, \end{array}$$

where, **V**_1_ is the sum of the volume of liver that is delineated by any of the 3 users, **V**_2_ is the sum of the volume that is delineated by any two of the 3 users, and **V**_3_ is the liver volume delineated by all the users. This can be pictorially expressed as in Figure 5.

## 3. Results

### 3.1. Segmentation Results

Comparison of the volumes obtained by the proposed algorithm in contrast to the manually calculated volumes, as provided in Table 2, shows an average volumetric error of 72.38 ± 61.46 mL (mean ± std. dev.) and an average absolute percentage error of 2.78 ± 4.39%. Table 2 also shows that an average Dice coefficient of 0.92 ± 0.01 is obtained for the proposed segmentation algorithm.

**Figure 6:**
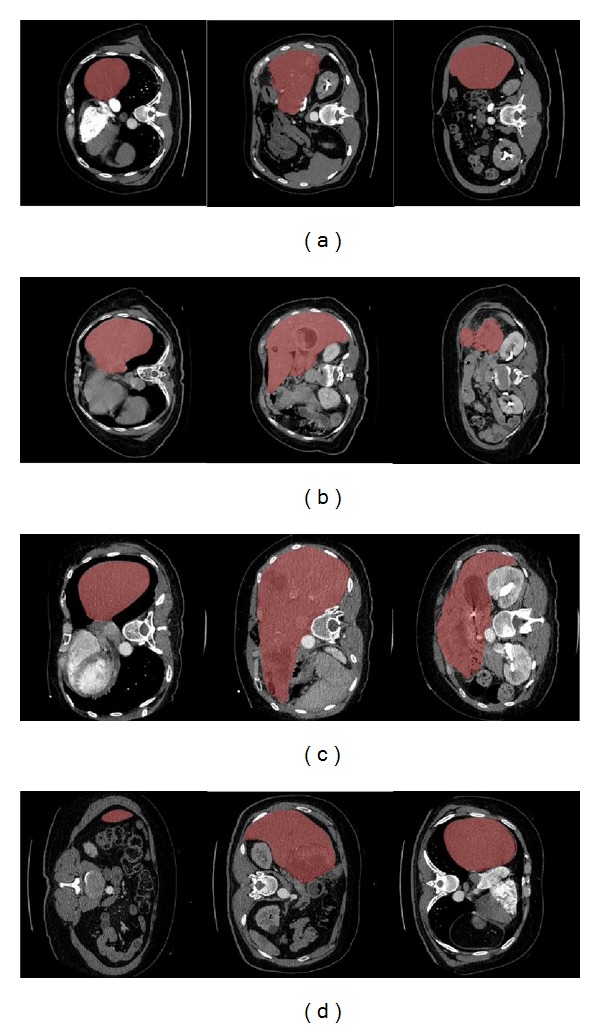
Illustrative examples of the segmentation results for 4 of the datasets: (a) Subject 4, (b) Subject 8, (c) Subject 12, and (d) Subject 17.

Figure 6 illustrates examples of the segmentation results (shown in brown) as obtained from 4 different datasets of the thirty-four used in the study. The slices are displayed in the range [−40 180] for the CT dataset for subjects 4, 8, 12, and 17 in Figures 6(a) to 6(d), respectively. The important point to be made is that there is a great variation in the intensity, structure, and position of the liver from dataset to dataset. Moreover, some of the datasets show the presence of tumors.

**Figure 7:**
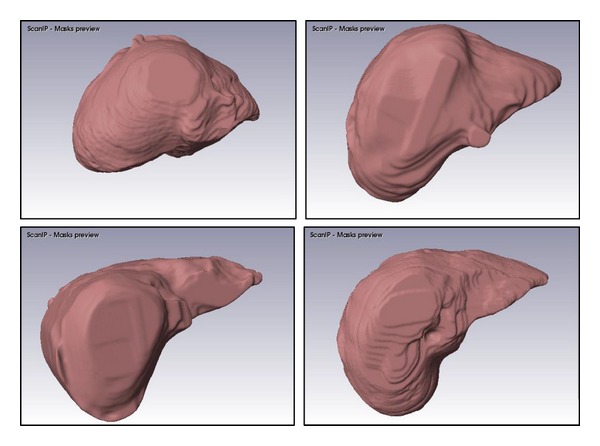
Illustrative examples of 3D renderings for 4 datasets used in the study.

Results of 3D rendering obtained from the ScanIP software are shown in Figure 7 for the different datasets. These 3D renderings are for the same datasets for which the slices were shown in Figure 6. The renderings shown here have solid surfaces and a mesh finish. However, translucent surfaces can be generated with varying opacities and colors if needed.

### 3.2. Statistical Analysis Results

The results of the ANOVA show that variations seen in the calculated volumes from multiple runs are not significantly different (*P* = 0.55), which establishes that the results of the proposed algorithm are consistent.

ANOVA analysis based on results obtained from multiple segmentations obtained from different user initializations shows that significant variations are not found among the segmentation outcomes (*P* = 0.23). The results of the statistical analysis suggest consistency of the segmentation algorithm in terms of multiple runs and different users.

Fleiss' kappa analysis showed a mean kappa of 0.77 ± 0.06, which is considered as a very good agreement among the segmentations found from the different initializations.

### 3.3. Comparisons with Other Liver Segmentation Algorithms

Table 3 displays the comparison between some of the current techniques found in the literature and the proposed technique in terms of the accuracy of the segmentation process and the computational aspects of the proposed algorithm. For the comparison of accuracy, the average volume difference between the calculated and the manual volumes is presented along with the standard deviation for the particular study.

For comparing the processing time, Table 3 compares the average computational time required to segment a single slice of the dataset obtained for the proposed study to various studies found in the literature. Such a comparison is essential since different algorithms use datasets with different number of slices for the analysis which determines the time needed for processing the entire dataset.

The table provides the interaction level of each algorithm where an interaction of less than 1 min is considered “low,” 1 to 5 min is considered “medium,” and greater than 5 min is considered “high.” An interaction level of “none” is displayed for automatic algorithms which are usually based on learning techniques and generally provide a lesser accuracy than interactive methods. The proposed algorithm falls in the low interaction category since it requires less than a minute to initialize the entire process.

## 4. Discussion

These results clearly demonstrate that the algorithm is highly accurate and consistent in calculating the volumetric measurements with a low standard deviation. Also, a maximum absolute percentage error of around 9% was recorded which falls within the clinical requirement of a maximum of 10%. Also, in some cases the error was as small as 0.36% (best case) for which the volumes between the segmented and manual gold standard calculations were almost the same.

From the results shown in Figure 6, it is observed that the algorithm correctly segments the liver region from the other organs in the body such as heart, stomach, spleen, and duodenum. The spleen and stomach are usually structures which are segmented incorrectly due to their similarity in the intensity and proximity to the liver. This difficulty was overcome with the novel contour initialization technique, which distinguishes the organs based on their location in the abdomen cavity.

The results given in Table 2 indicate that the liver segmentation algorithm provides highly accurate results for livers of different sizes ranging from 798 mL to 5336 mL. This is an important feature for any liver segmentation approach to be applied for cancer treatment and especially SIRT since the size of the liver is greatly affected in diseased situations. Also, the algorithm is shown not to be affected by cases where a resection has been performed in a previous intervention. One such case has been displayed in Figure 6(a). This makes the algorithm practical for posttreatment followup as well.

Although accurate volumetric measurements are sufficient in SIRT dosing calculation, the Dice coefficient results provide the degree of accuracy for independent slice segmentations. This is essential to be shown since volumetric measures are sometimes deceptive as an increase in volume in one region may be compensated by a decrease in volume in another region and vice versa. It was determined for the study that an average Dice coefficient of 0.92 was obtained throughout the datasets. Such a high Dice coefficient clearly demonstrates the merits of the algorithm.

It should also be noted that the span of the liver is different for the different datasets. In most cases, the liver shows the usual anatomy of a triangular organ with most of its mass on the right side of the body descending inferiorly towards the right kidney (e.g., Figures 6(a) and 6(b)). However, in other cases the liver spans the entire abdominal cavity just inferior to the diaphragm (see Figure 6(c)). The algorithm is still capable of extracting the liver correctly with an accuracy of less than 10%, which is considered as the clinical error threshold for critical applications like SIRT. This is an advantage of the segmentation algorithm over model-based and learning-based approaches, which fail in situations where such large deviations from “normal” structures are seen. Also, since the algorithm does not need to be trained like in model-based approaches, independent analysis is reinforced and influence of one dataset over others is removed. Another interesting feature evident from Figure 6(d) relates to situations where the image data is rotated such that the patient data is obtained as if the patient was scanned feet first instead of head-first. The proposed algorithm did not fail under such a circumstance since it is not dependent on prior knowledge of the dataset under consideration.

A high accuracy in the determination of the liver volume is essential for SIRT studies since the radiation dose to be delivered to the patient is determined relative to the patient's liver volume. This reduces the risk of excess dosing, which may damage healthy tissue or reduced dosing which may result in a tumor relapse.

The statistical analysis provided in the results section demonstrated that the segmentation process provides consistent results when carried out multiple times on the same dataset (*P* > 0.05). Also, it was shown that the results of the segmentation were not dependent on the user initialization of the algorithm (*P* > 0.05) for which 3 different users were asked to initialize the segmentation task. Such a feature is essential for an algorithm since various individuals ranging from scientists to physicians may at any point require using the algorithm.

The algorithm fared well with respect to the computational burden imposed by the subtle intricacies of the algorithm itself as well as the amount of imaging data required in processing each 3D CT dataset. The algorithm as designed is parallel-aware and can be deployed on larger computer clusters if the need arises to reduce significantly the processing time to seconds. Although, the comparison process provided encouraging results, it should be noted that the different studies used for the comparison process used different datasets and hardware systems for the deployment of the algorithm. Also, in addition to the different datasets and hardware configurations the various algorithms listed in the comparison use different programming languages such as C, C++, Java, Visual Basic, and MatLab.

In retrospect the study provided a novel liver segmentation paradigm with very low human interaction in the form of a single slice being initialized by the user.

## 5. Conclusion

The study demonstrated the development of a novel robust and accurate liver segmentation technique using very low human interaction. The developed algorithm performed the segmentation integrating a modified k-means algorithm with a localized contouring algorithm. A novel contour initialization technique based on region growing and volume of interest based correction was developed. The proposed algorithm achieved the entire segmentation through the initialization of a single slice.

The algorithm demonstrated a very high accuracy of 97.22% in determining the liver volumes. Also, average Dice coefficient of 0.92 was obtained for the study demonstrating the high accuracy of the segmentation process. Statistical analysis proved that the results obtained were highly consistent (*P* = 0.55) and that the segmentation process was independent of user initialization (*P* = 0.23 and Fleiss' Kappa = 0.77 ± 0.06).

Since the radioactive dose to be delivered to the patient relies on the tumor to liver volume ratio, accurate calculation of the liver volume would help in the determination of a more precise radioactive dosage to the patient, avoiding as a consequence underdosing resulting in recurrence of tumor or overdosing resulting in damage to healthy tissue.

## Figures and Tables

**Table 1:** Profiles of the users initializing the segmentation process.

User	Knowledge of liver anatomy	Knowledge of algorithms/programs	Occupation
1	5	1	Medical doctor/oncologist
2	3	2	Biomedical engineer
3	1	4	Electrical engineer

**Table 2:** Comparative results between the proposed algorithm and manual gold standard.

Number	Manual segmentation volumes (mL)	Average calculated volumes (mL)	Absolute error (mL)	% Error	Average Dice coefficient
1	1458.80	1426.00	32.80	−2.25	0.94
2	4079.50	4180.67	101.17	2.48	0.92
3	2419.18	2402.00	17.18	−0.71	0.90
4	1848.73	1855.33	6.60	0.36	0.90
5	1630.59	1649.33	18.74	1.15	0.91
6	1568.82	1448.67	120.15	−7.66	0.91
7	1408.27	1358.67	49.60	−3.52	0.94
8	1331.81	1318.00	13.81	−1.04	0.94
9	2595.61	2637.33	41.72	1.61	0.92
10	2651.20	2709.67	58.47	2.21	0.91
11	1631.40	1703.33	71.93	4.41	0.92
12	2892.98	2626.67	266.31	−9.21	0.90
13	1904.25	1966.00	61.75	3.24	0.93
14	2704.79	2832.67	127.87	4.73	0.93
15	1602.26	1545.67	56.59	−3.53	0.93
16	1565.97	1491.67	74.30	−4.74	0.93
17	2408.85	2376.00	32.85	−1.36	0.90
18	5336.28	5180.33	155.95	−2.92	0.93
19	1102.16	1139.33	37.17	3.37	0.91
20	2363.25	2466.00	102.75	4.35	0.94
21	1202.18	1102.57	99.61	−8.29	0.92
22	1801.65	1681.30	120.35	−6.68	0.93
23	1749.51	1647.10	102.41	−5.85	0.93
24	1558.69	1540.53	18.16	−1.17	0.95
25	1797.16	1642.74	154.42	−8.59	0.92
26	1749.79	1709.54	40.25	−2.30	0.94
27	1017.20	974.51	42.69	−4.20	0.92
28	942.02	879.72	62.30	−6.61	0.91
29	2248.62	2062.40	186.22	−8.28	0.91
30	1006.68	944.81	61.87	−6.15	0.92
31	798.90	753.67	45.23	−5.66	0.92
32	1591.80	1449.90	141.90	−8.91	0.92
33	2072.80	1975.49	97.31	−4.69	0.95
34	1753.76	1614.09	139.68	−7.96	0.92

Avg.	**1935.16**	**1890.93**	**81.18**	**−2.78**	**0.92**
Std. dev.	**895.08**	**901.41**	**57.36**	**4.39**	**0.01**

**Table 3:** Comparison of proposed algorithm with other algorithms found in the literature.

Method	Interaction level	Volume difference (% error)	Processing time per slice (sec)
Beck and Aurich (see [40])	*high *	1.8 ± 2.5	3.00
Beichel et al. (see [41])	*high *	1.0 ± 1.7	15.43
Chi et al. (see [30])	*none *	2.6 ± 6.3	14.57
Dawant et al. (see [42])	*medium *	2.5 ± 2.3	8.57
Furukawa et al. (see [20])	*none *	−7.3 ± 4.7	15.43
Goryawala et al. (see [47])	*medium *	1.7 ± 2.1	7.50
Heimann et al. (see [25])	*none *	1.7 ± 3.2	3.00
Kainmüller et al. (see [14])	*none *	−2.9 ± 2.9	6.43
Lee et al. (see [43])	*low *	1.3 ± 2.9	3.00
Rusko et al. (see [34])	*none *	−3.8 ± 6.4	0.21
Saddi et al. (see [26])	*none *	1.2 ± 4.4	2.36
Schmidt et al. (see [41])	*none *	−4.9 ± 3.0	8.57
Seghers et al. (see [17])	*none *	−6.8 ± 2.3	12.86
Susomboon et al. (see [44])	*none *	−11.5 ± 30	10.71
van Rikxoort et al. (see [21])	*none *	1.8 ± 4.2	19.29

Proposed algorithm	***low***	2.78 ± 4.39	**10.96**
